# Supplementary material for: A metallic molybdenum dioxide with high stability for surface enhanced Raman spectroscopy
Source: Nat Commun. 2017 Apr 24;8:14903. doi: 10.1038/ncomms14903 (PMC5413975; doi:10.1038/ncomms14903)
Supplement: Supplementary Information — Supplementary figures, supplementary table, supplementary methods and supplementary references. [file ncomms14903-s1.pdf]

Supplementary methods

Enhanced Factor Calculation

To calculate the EF of the MoO_2 nanodumbbells, the ratio of SERS to normal Raman spectra (NRS) of Rh6G was determined by using the following calculating formula 1

$EF = (I_{SERS}/N_{SERS})/(I_{NRS}/N_{NRS})$ (1)

$N_{SERS} = N_A n S_{Irr}/S_{dif}$ (2)

$N_{NRS} = dS_{Irr}hN_A/M$ (3)

where I_{SERS} and I_{NRS} refer to the peak intensities of the SERS and NRS, respectively. N_{SERS} and N_{NRS} correspond to the number of probe molecules excited in the SERS and NRS tests. In the SERS measurements, two Raman scattering peaks, R_1 at 612 cm^{-3} and R_2 at 773 cm^{-3} were selected for the calculations of the EF. For comparison, the peak intensities of the Rh6G (1×10^{-2} M, aqueous solution) directly placed on bare glass were detected as NRS data. To decrease the measuring error, the intensities were obtained by continually ran the test procedure at randomly selected 40 points and took the average. N_{SERS} is calculated by formula 2, where N_A refer to the Avogadro's constant, n correspond to the molar quantity of the probe molecule, S_{Irr} refer to the irradiation area under the laser beam (5 μm in diameter), and S_{dif} refer to the diffusion area of the substance to be tested on the substrate. In a typical test, one drop (20 microliter) of the probe solution was dropped onto the SERS substrate, and the probe solution was spread into a circle with a diameter of 4 mm when the solution is completely dry. N_{NRS} is determined by the formula 3, where d is the packing density of Rh6G molecules in the surface of substrate (1.4×10^{21} molecule/cm^3), h refer to the

laser confocal depth (26 μm), M correspond to the molecule weight of Rh6G (479).

Calculations details

All density functional theory (DFT) calculations were carried out using the Vienna Ab initio Simulation Package (VASP)[1-3]. The generalized gradient approximation of Perdew-Burke-Ernzerhof (GGA-PBE)[4] was used to describe the exchange-correlation functional. A plane-wave basis set with an energy cutoff of 400eV was used to expand the one-electron wave function, and the Brillouin zones were sampled with Monkhorst–Pack[5] k point meshes of 5 × 6 × 5, 13 × 3 × 13 for MoO_2 and MoO_3, respectively. The geometry optimization was stopped when the forces on each atom were less than 0.02 eV/Å. The optimized lattice parameters were 5.58, 4.89, 5.64 Å for MoO_2 and 4.04, 13.85, 3.77 Å for MoO_3, which agree well with previous experimental and calculated results[6,7].

Synthesis of urchin-like $W_{18}O_{49}$ nanowires

This synthetic method is referred to the literature reported by Ye group[8]. In a typical procedure, 2 g of WCl_6 was dissolved in 100 mL of ethanol, and the obtained yellow solution was magnetically stirred for 2 h, and then transferred to a Teflon–lined stainless–steel autoclave and heated at 180 °C for 20 h with a heating rate of 4 °C/min. The autoclave was cool down naturally and a blue product was collected, washed, and dried in a vacuum drying oven at 50 °C for 4 h.

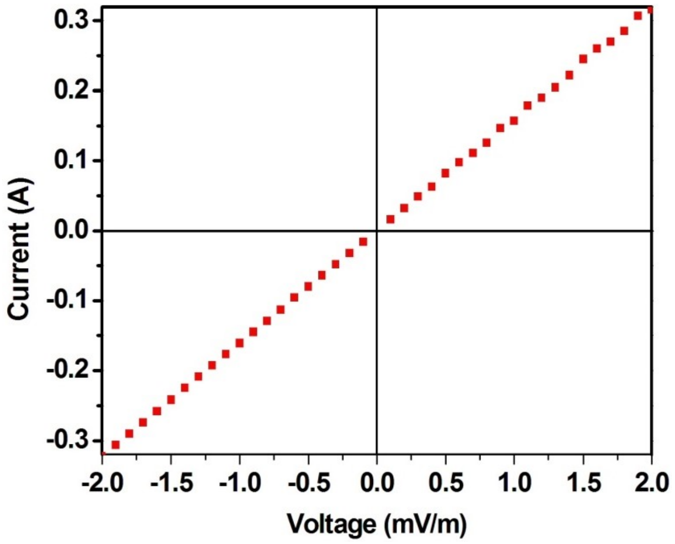

Supplementary Figure 1. Room-temperature I-V behaviors of the MoO_2 sample.

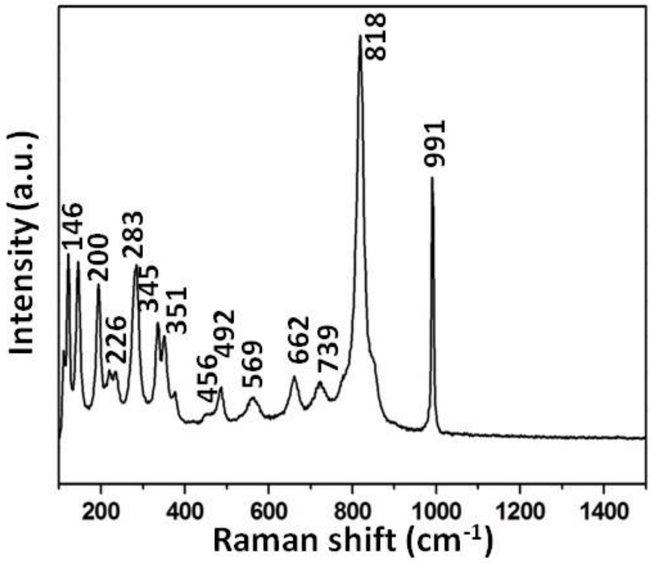

Supplementary Figure 2. Raman spectrum of the as-synthesized MoO_2 nanodumbbells.

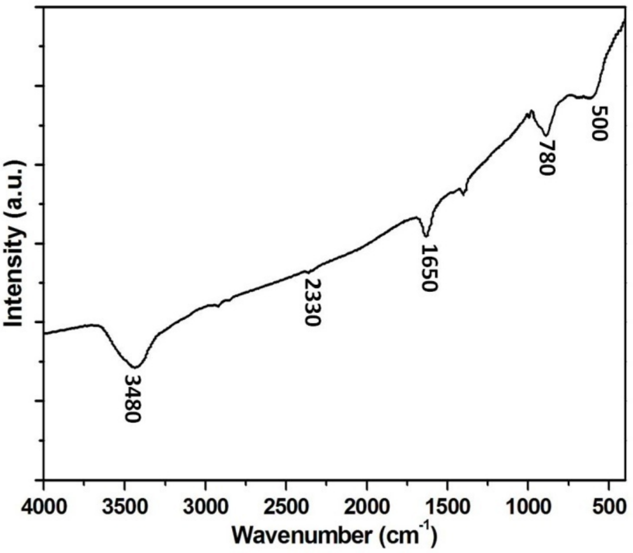

Supplementary Figure 3. FTIR spectrum of the as-synthesized MoO_2 nanodumbbells.

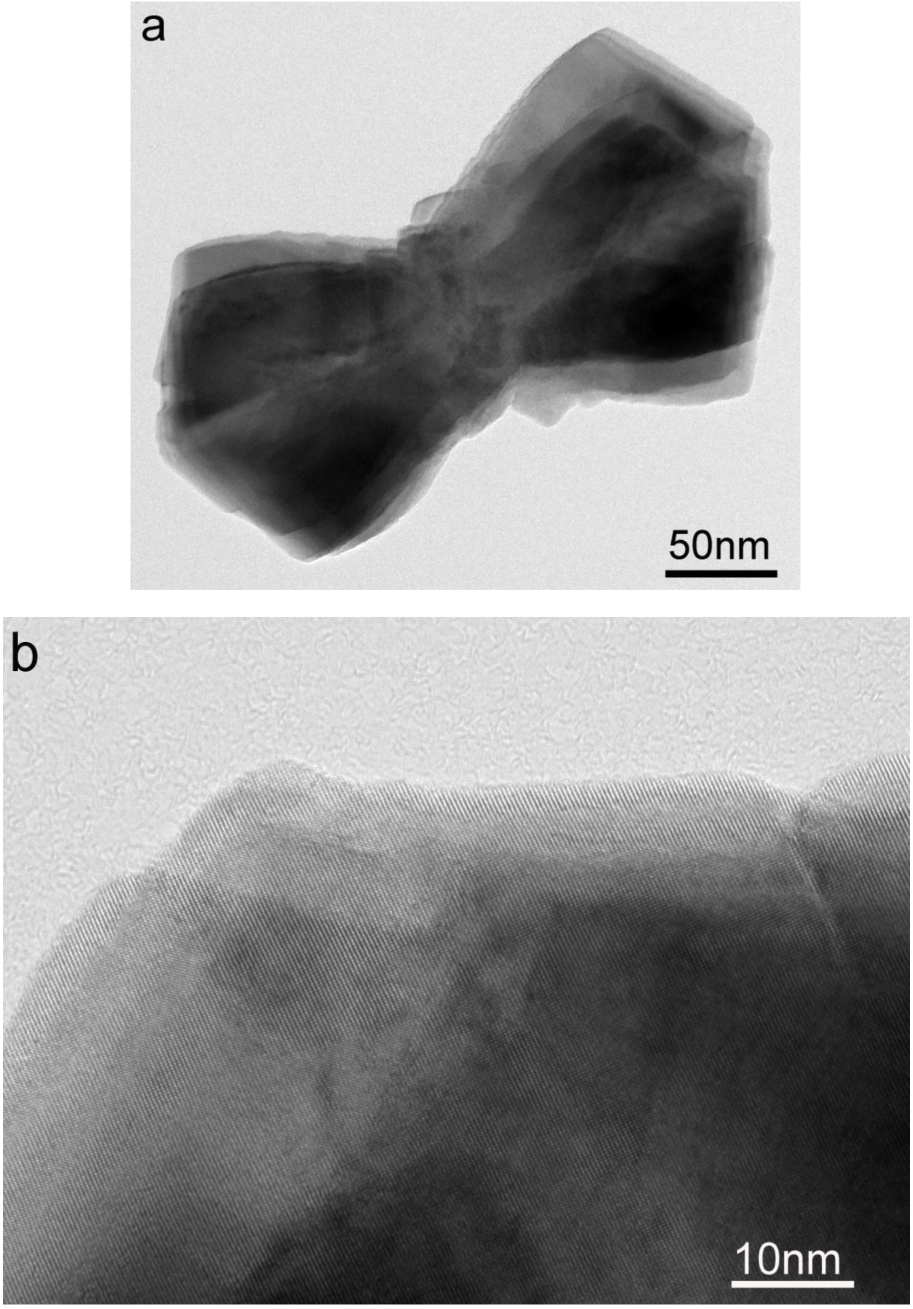

Supplementary Figure 4. a, TEM image of a complete MoO_2 nanodumbbell, which clearly indicates that the dumbbell-like nanostructure is composed of many layers of thin nanosheets. **b,** HRTEM image of the MoO_2 nanodumbbell, which demonstrates that each layer of the nanosheets is highly crystalline.

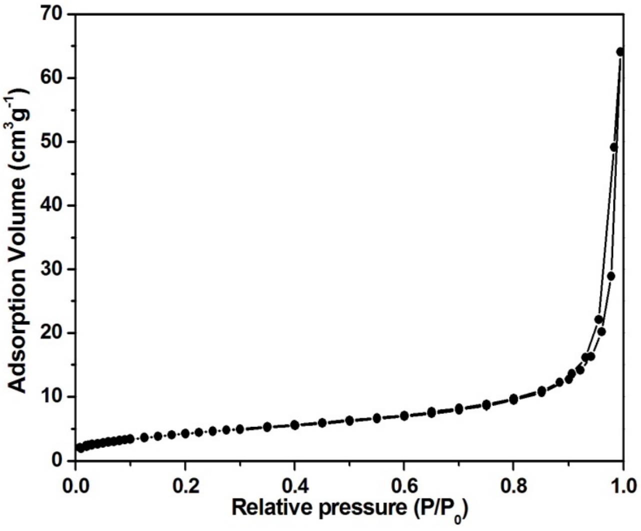

Supplementary Figure 5. N_2 adsorption/desorption isotherms of the as-synthesized MoO_2 nanodumbbells.

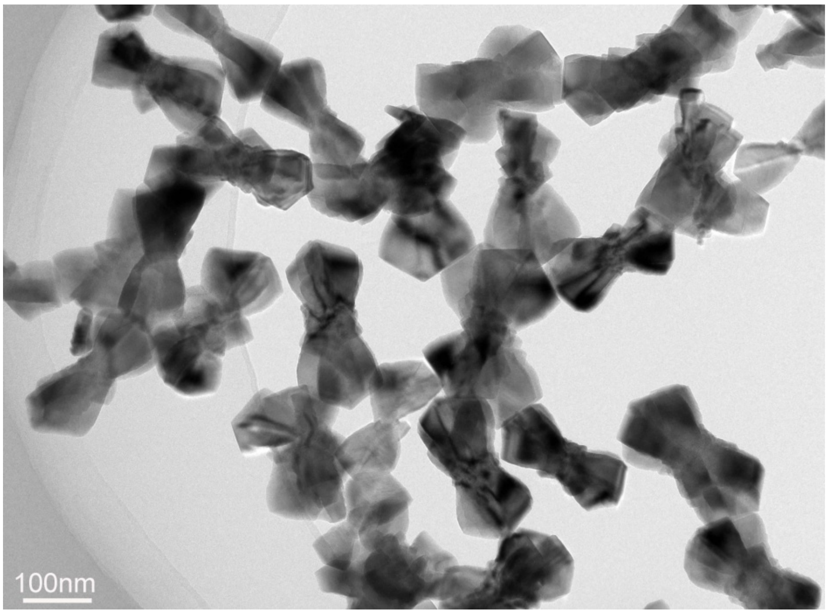

Supplementary Figure 6. TEM image of the MoO_3 nanodumbbells prepared by heating the MoO_2 nanodumbbells at 600 °C.

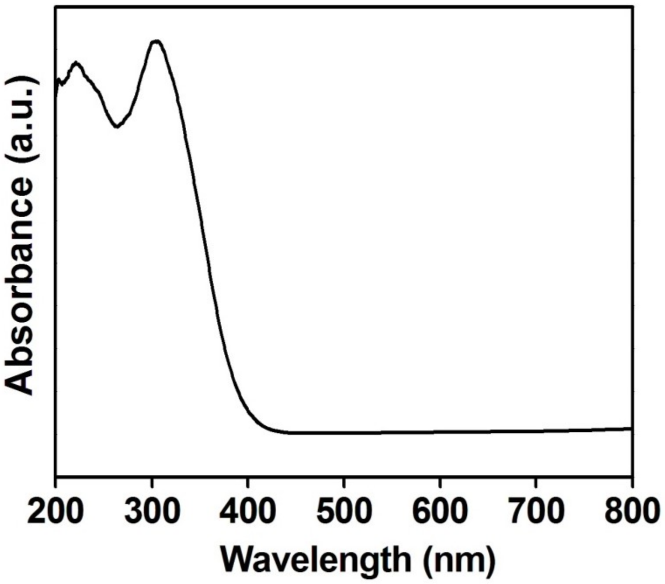

Supplementary Figure 7. UV-Vis absorption spectrum of the MoO_3 nanodumbbells prepared by heating the MoO_2 nanodumbbells at 600 °C.

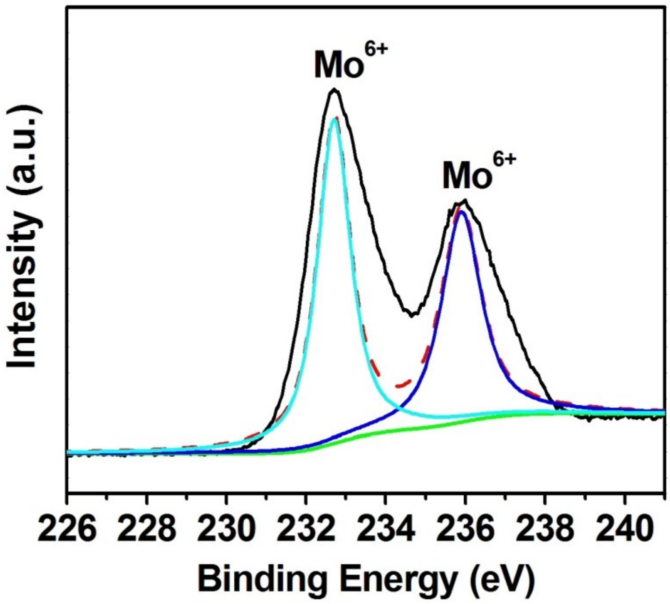

Supplementary Figure 8. XPS spectrum of the MoO_3 nanodumbbells prepared by heating the MoO_2 nanodumbbells at 600 °C.

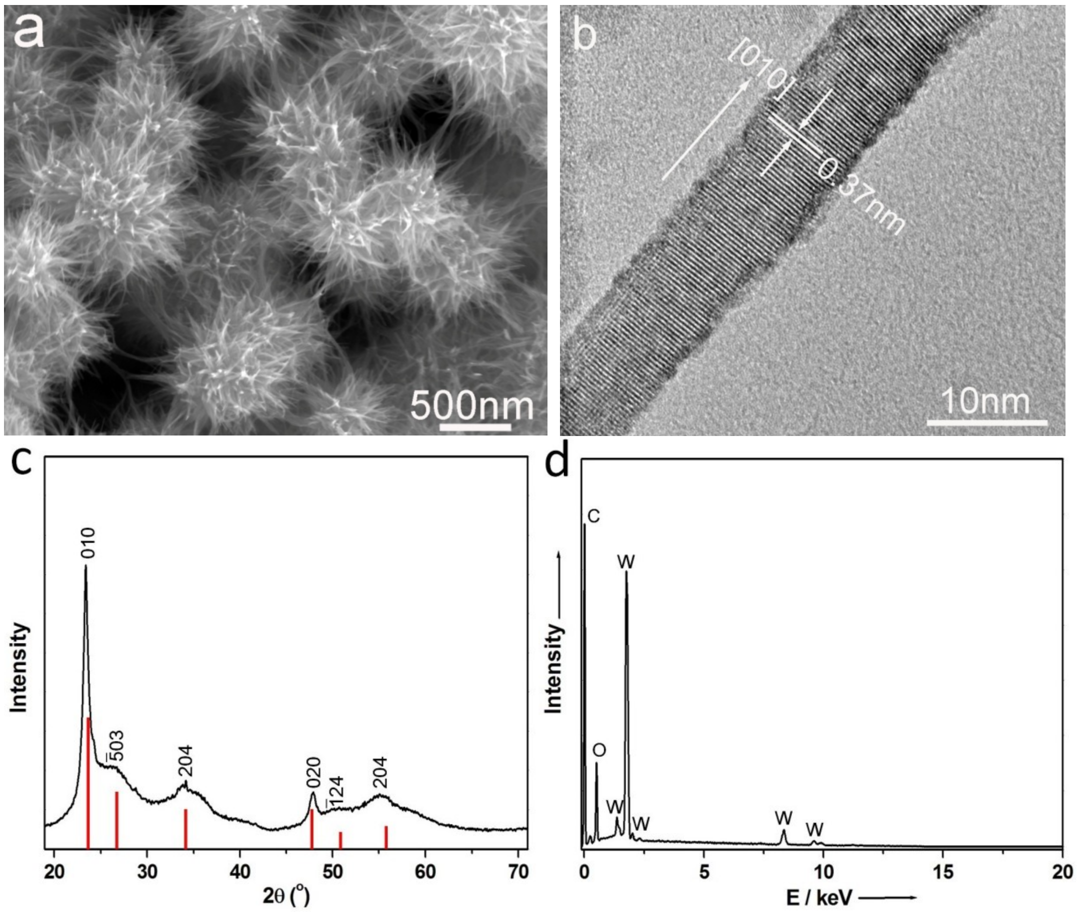

Supplementary Figure 9. Characterization of the as-synthesized $W_{18}O_{49}$. a-b, SEM image and HRTEM image of the prepared urchin-like $W_{18}O_{49}$ nanowires. The HRTEM indicates that the growth direction of the $W_{18}O_{49}$ nanowires is [010]. **c,** XRD pattern of the sample can be indexed with monoclinic $W_{18}O_{49}$ (JCPDS No.: 05–0392). The narrow (010) and (020) peaks strongly suggest that the possible crystal growth direction of the sample is [010], since the close-packed planes of the monoclinic $W_{18}O_{49}$ crystal are {010}, which are highly consistent with the HRTEM characterization results. **d,** EDS elemental analysis of the sample.

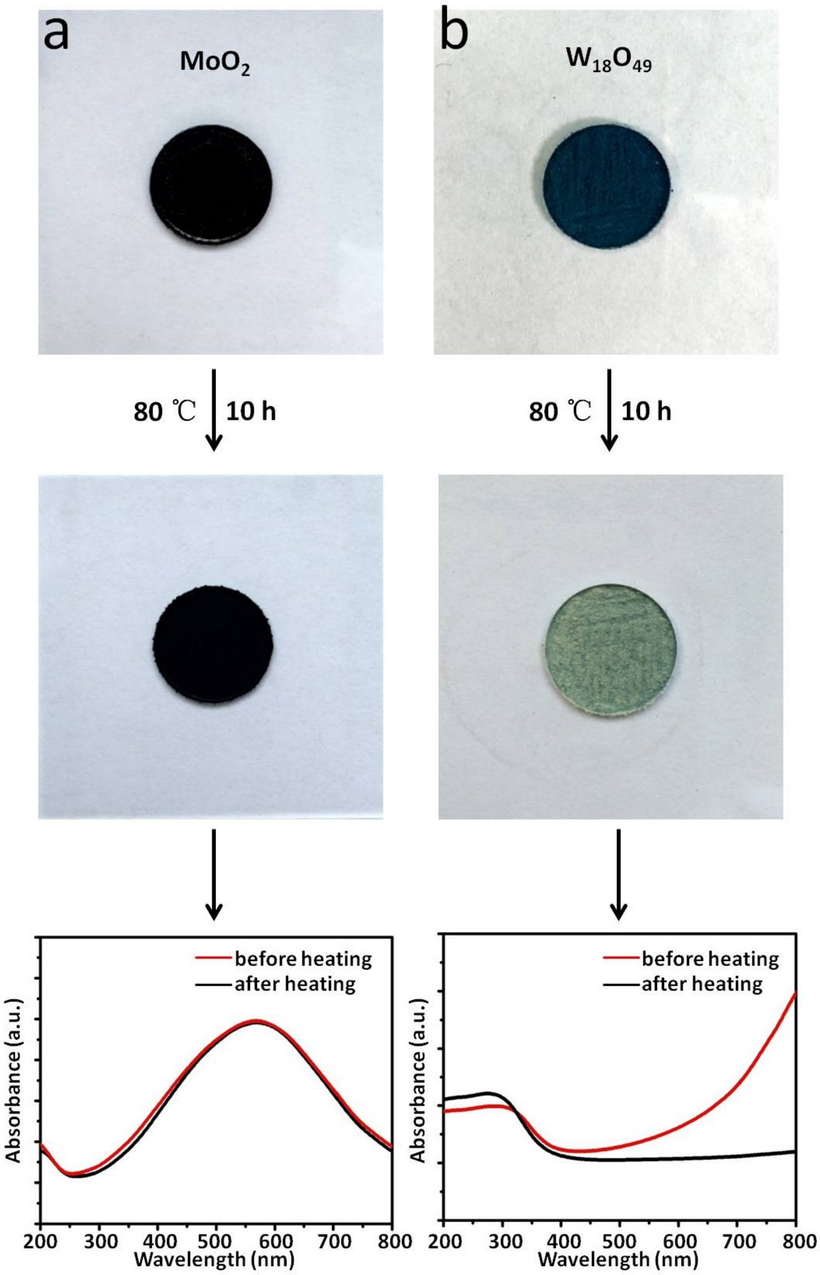

Supplementary Figure 10. The comparison of oxidation resistance between MoO_2 and $W_{18}O_{49}$. The change of color and absorbance before and after heating from two kinds of samples clearly indicated that the oxidation resistance of $W_{18}O_{49}$ is much lower than that of MoO_2.

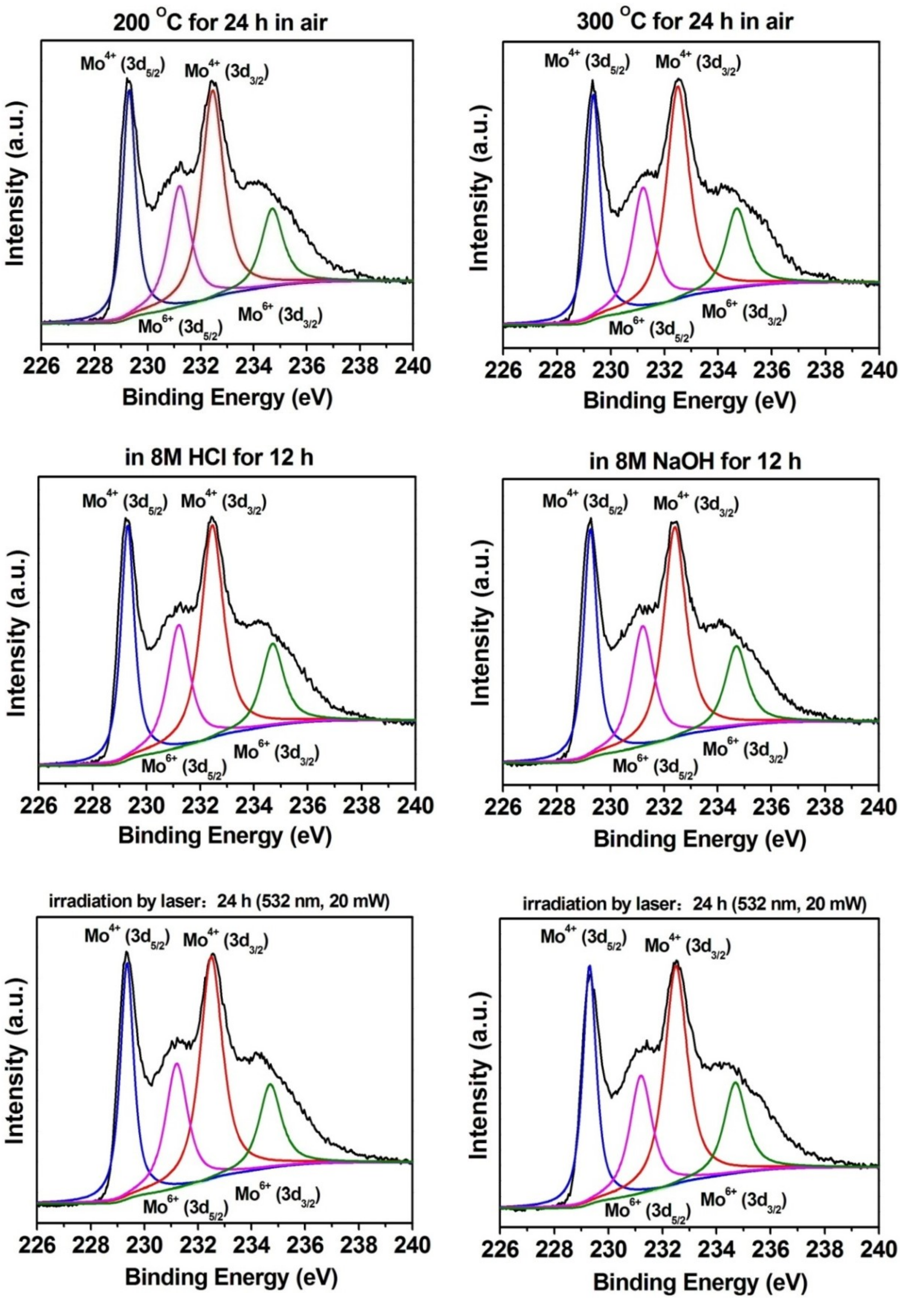

Supplementary Figure 11. A series of XPS spectra demonstrated that no detectable change in the surface valence states of Mo in the MoO_2 nanodumbbells after the heating, irradiating, acid and alkali corroding. The XPS results demonstrated that the stability of these MoO_2 nanodumbbells is extraordinary high.

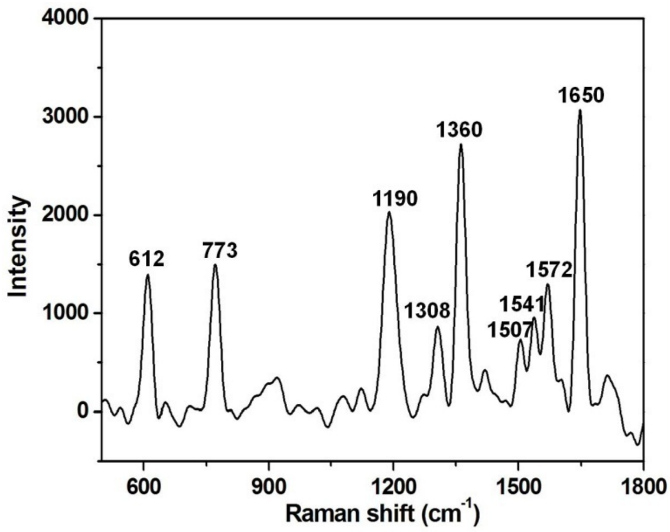

Supplementary Figure 12. The Raman spectrum of the reference material of Rh6G.

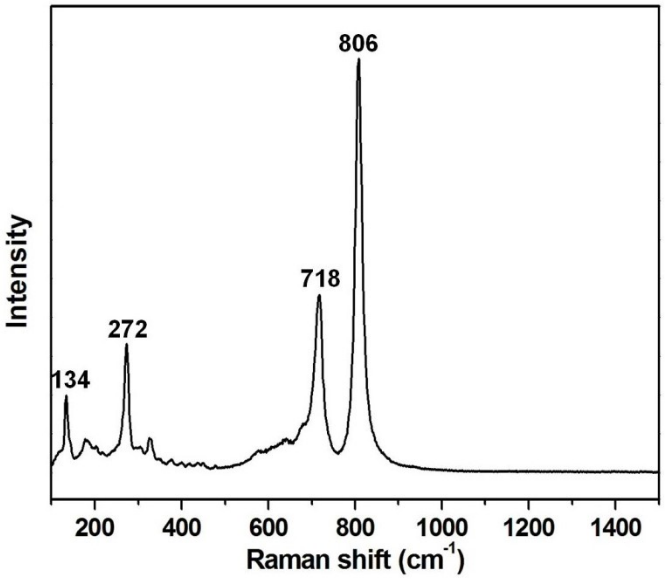

Supplementary Figure 13. Raman spectrum of 10^{-4} M Rh6G aqueous solution obtained in tungsten oxide nanowires heated at 80 °C for 10 h. No Raman scattering signal of Rh6G was detected, and only the typical Raman scattering signals of tungsten oxide was observed, which suggests that the stability of the $W_{18}O_{49}$ nanowires is poor.

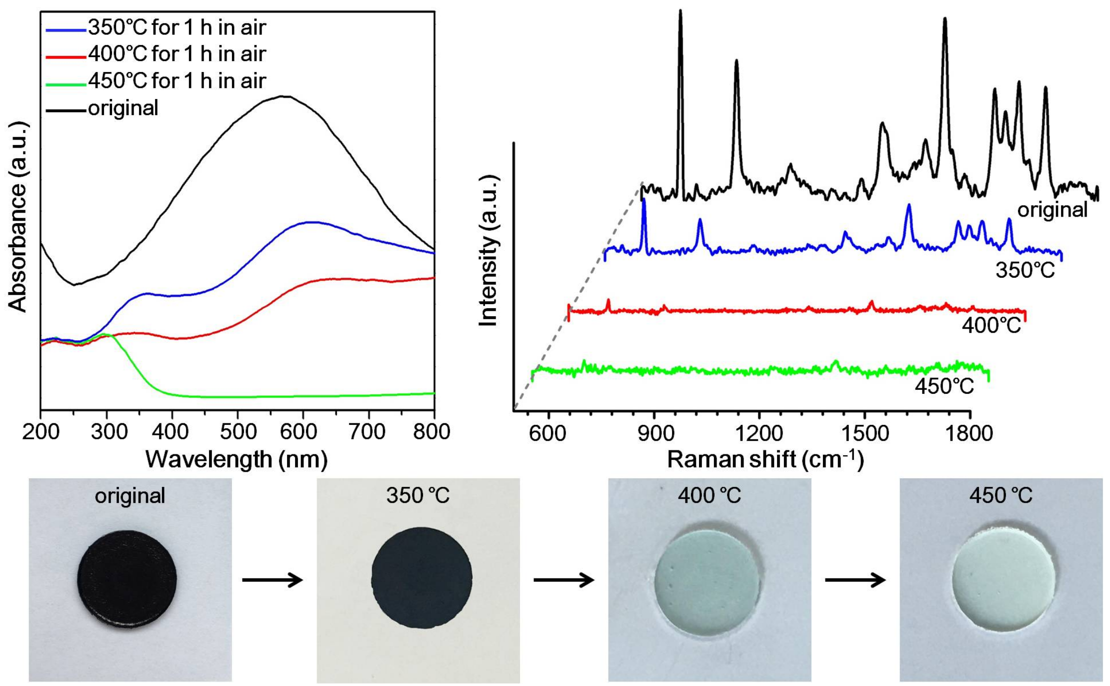

Supplementary Figure 14. When the samples were heated at 350, 400, 450 °C for 1 h in air, with the increase of oxidation state and free electron density decreased, the plasma resonance absorption peak of them decreased violently (left-above figure). Accordingly, their corresponding SERS performance is also greatly decreased (right-above figure).

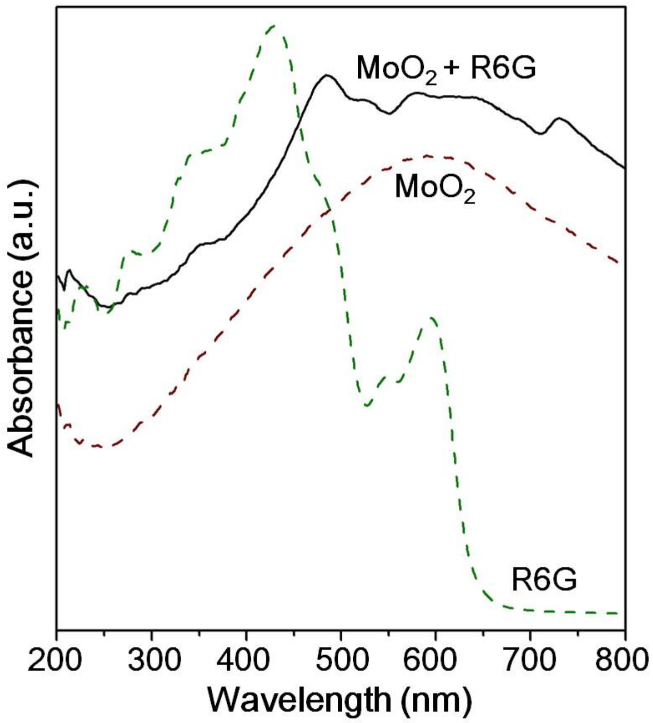

Supplementary Figure 15. UV-Vis absorption spectra for R6G-modified MoO_2 compared with neat MoO_2 and R6G.

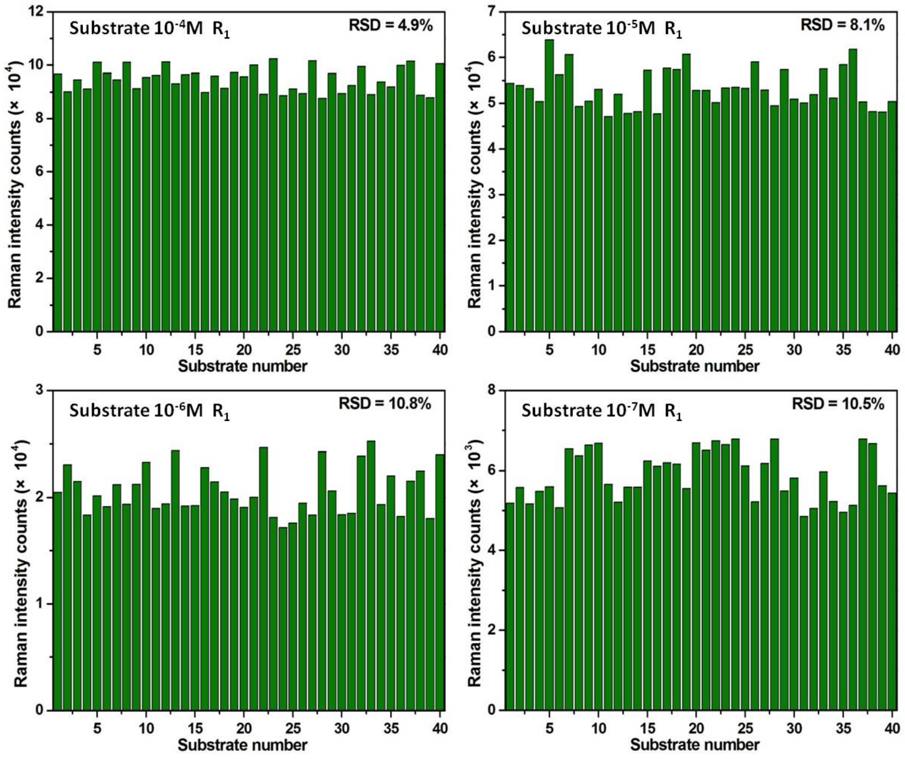

Supplementary Figure 16. Relative standard deviation (RSD) of statistically obtained from more MoO_2 substrates by using the intensities of R_1. For each concentration, a total of 40 intensity values were collected from 8 MoO_2 substrates, that is, 5 measurement points were randomly taken on each substrate.

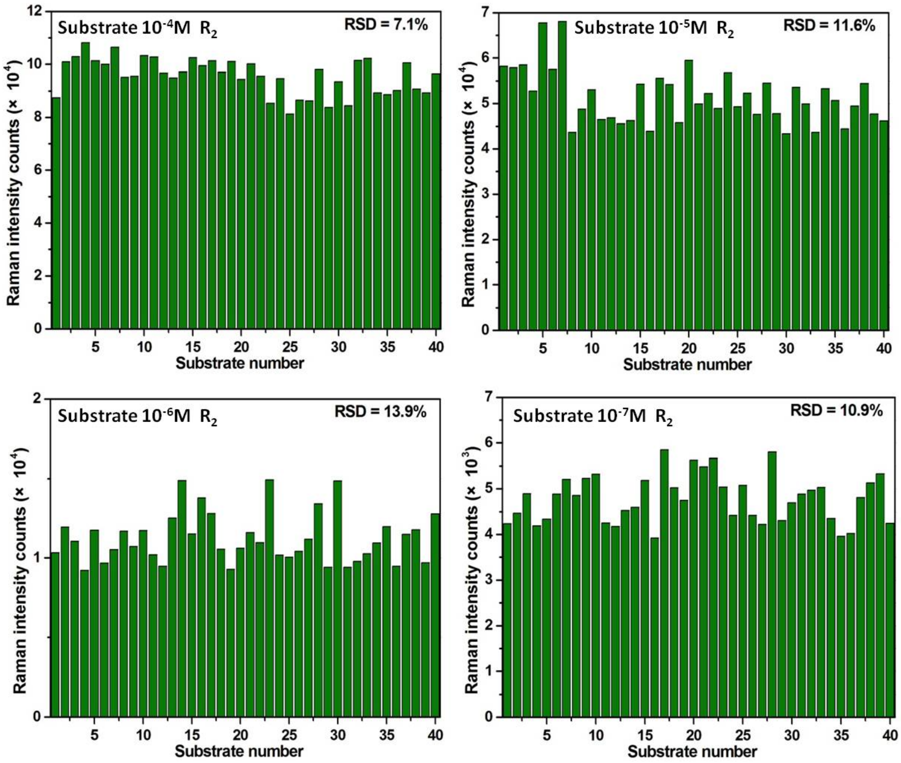

Supplementary Figure 17. Relative standard deviation (RSD) of statistically obtained from more MoO_2 substrates by using the intensities of R_2. For each concentration, a total of 40 intensity values were collected from 8 MoO_2 substrates, that is, 5 measurement points were randomly taken on each substrate.

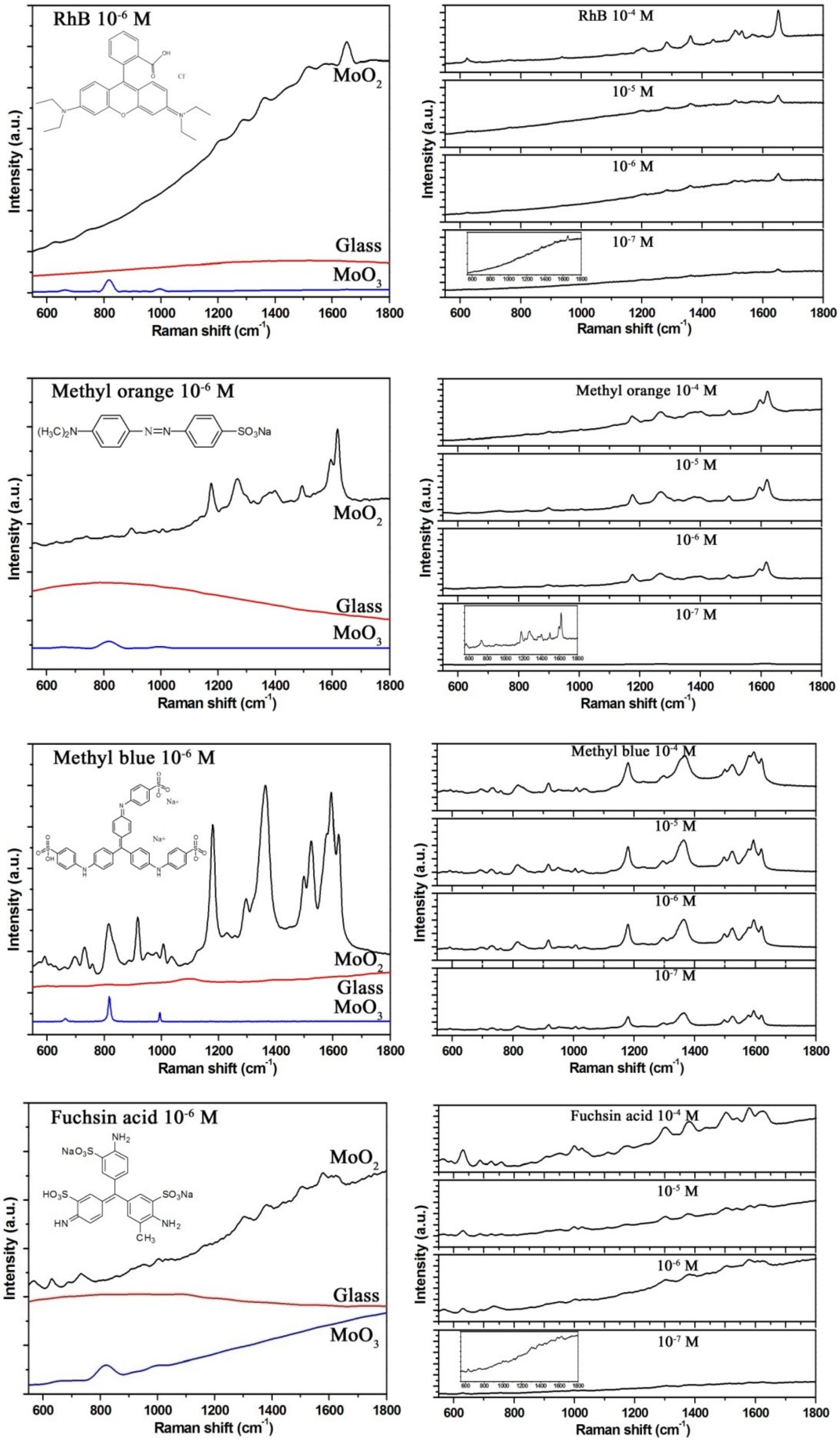

Supplementary Figure 18. SERS spectra of a series of dye molecules: RhB, MO, MB, and FA.

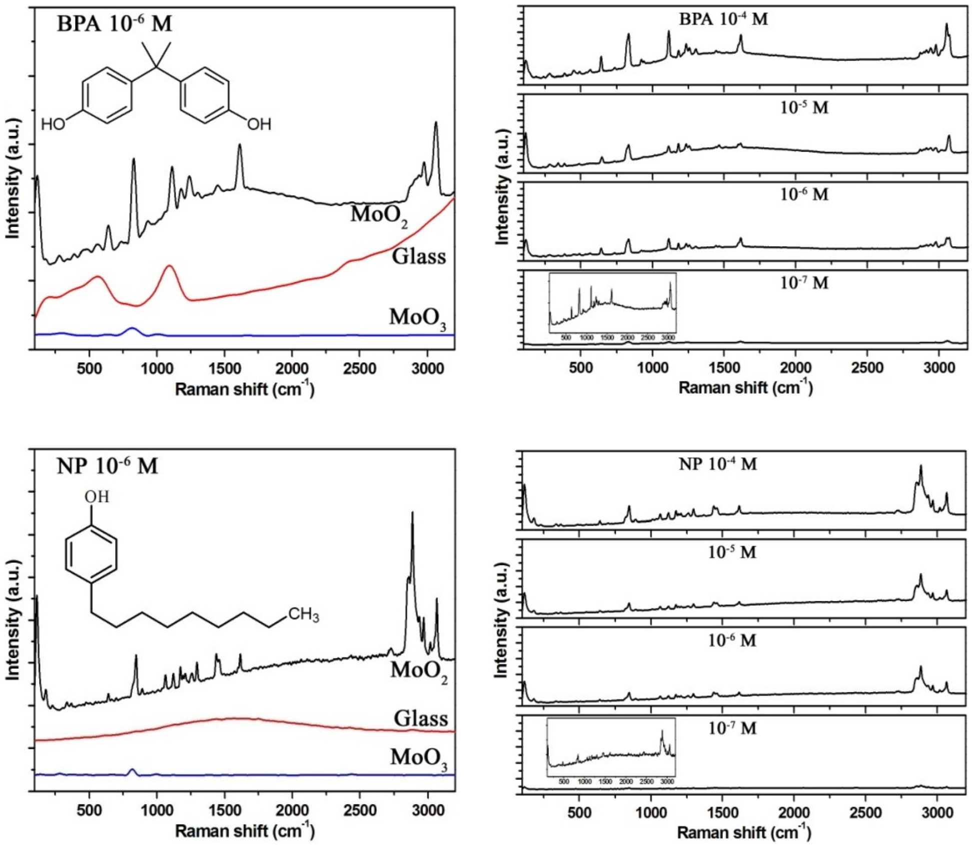

Supplementary Figure 19. SERS spectra of Bisphenol A (BPA) and nonyl phenol (NP).

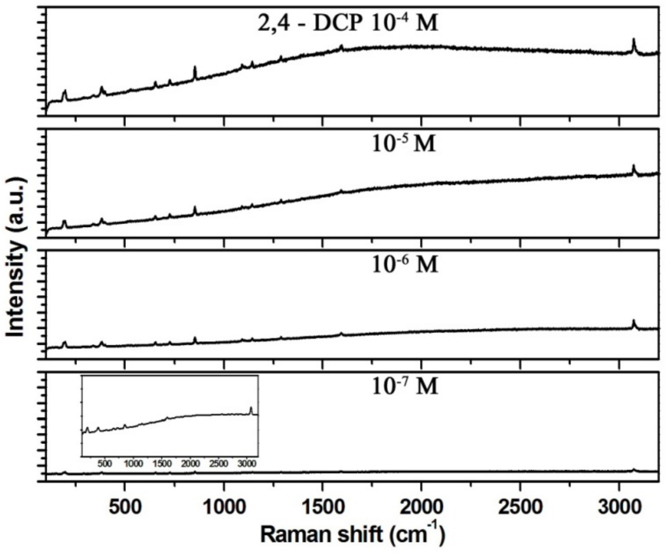
2,4 - DCP 10-4 M
10-5 M
10-6 M
10-7 M
Intensity (a.u.)
500
1000
1500
2000
2500
3000
Raman shift (cm-1)

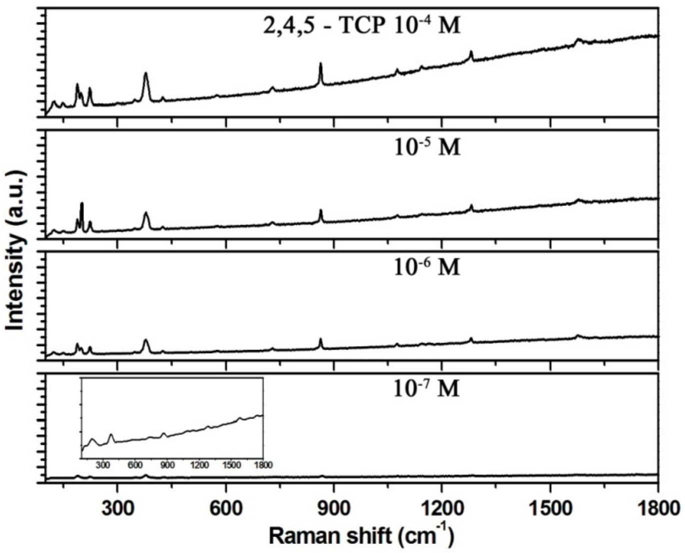
2,4,5 - TCP 10-4 M
10-5 M
10-6 M
10-7 M
Intensity (a.u.)
300
600
900
1200
1500
1800
Raman shift (cm-1)

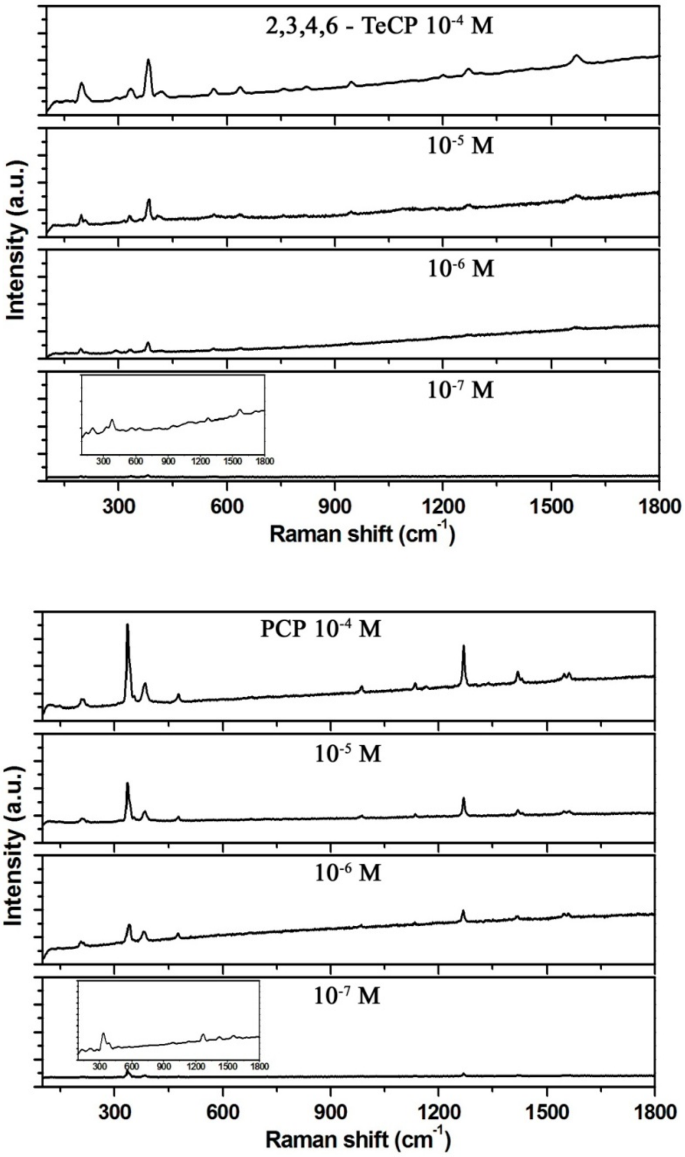

Supplementary Figure 20. Gradually weakened Raman scattering signals recorded from 2,4-DCP, 2,4,5-TCP, 2,3,4,6-TeCP, and PCP aqueous solution at four different concentration levels (10^{-4}, 10^{-5}, 10^{-6}, 10^{-7} M).

Supplementary Table 1. Enhancement factors （EFs) for various materials have been reported.

Material	Probe molecule	**EF**	Excited wavelength (nm)	Author
Au metamaterial	benzenethiol	**2.62×10^5**	782	S. Y. Zhang et al[9].
Ag on porous Si	R6G	**10^5**	488	H. H. Lin et al[10].
Ag nanoparticles	MGITC	**10^7**	632.8	H. Y. Liang et al[11].
Au–CdSe nanowires	CV	**10^4**	633	G. Das et al[12].
CdS	4-Mpy	**10^2**	514.5	Y. F. Wang et al[13].
CdTe	4-Mpy	**10^4**	514.5	Y. F. Wang et al[14].
Colloida ZnO	D266	**50**	488	H. Wen et al[15].
TiO_2 photonic microarray	MB	**2×10^4**	532	D. Qi et al[16].
TiO_2–Ag core–shell material	4-Mpy	**6.5×10^5**	514	X. X. Zou et al[17].
$\alpha-Fe_2O_3$	4-Mpy	**2.7×10^4**	514.5	X. Q. Fu et al[18].
Cu_2O	4-MBA	**10^5**	488	L. Jiang et al[19].
CuO	4-Mpy	**10^2**	514.5	Y. Wang et al[20].
$W_{18}O_{49}$	R6G	**3.4×10^5**	532.8	S. Cong et al[21].
**MoO_2**	**R6G**	**3.75×10^6**	**532**	**this work**

Supplementary References

1. Kresse, G. & Hafner, J. Ab initio molecular-dynamics simulation of the liquid-metal–amorphous-semiconductor transition in germanium. *Physical Review B* **49**, 14251-14269 (1994).

2. Kresse, G. & Furthmüller, J. Efficiency of ab-initio total energy calculations for metals and semiconductors using a plane-wave basis set. *J. Computational Materials Science* **6**, 15-50 (1996).

3. Kresse, G. & Furthmüller, J. Efficient iterative schemes for ab initio total-energy calculations using a plane-wave basis set. *Physical Review B* **54**, 11169-11186 (1996).

4. Perdew, J. P. Burke, K. & Ernzerhof, M. Generalized gradient approximation made simple. *Physical Review Letters* **77**, 3865-3868 (1996).

5. Monkhorst, H. J. & Pack, J. D. Special points for Brillouin-zone integrations. *Physical Review B* **13**, 5188-5192 (1976).

6. Scanlon, D. O. Watson, G. W. Payne, D. J. Atkinson, G. R. Egdell, R. G. & Law, D. S. L. Theoretical and experimental study of the electronic structures of MoO_3 and MoO_2. *The Journal of Physical Chemistry C* **114**, 4636-4645 (2010).

7. Seisenbaeva, G. A. Sundberg, M. Nygren, M. Dubrovinsky, L. Kessler, V. G. Thermal decomposition of the methoxide complexes $MoO(OMe)_4$, $Re4O_6(OMe)_{12}$ and $(Re_{1-x}Mo_x)O_6(OMe)_{12}$ ($0.24 \leq x \leq 0.55$). *Mater. Chem. Phys.* **87**, 142-148 (2004).

8. Xi, G. C. Ye, J. H. Ma, Q. Su, N. Bai, H. & Wang, C. In situ growth of metal particles on 3D urchin-like WO_3 nanostructures. *J. Am. Chem. Soc.* **134**, 6508–6511 (2012).

9. Zhang, S. Y. et al. Hierarchical porous plasmonic metamaterials for reproducible ultrasensitive surface-enhanced Raman spectroscopy. *Adv. Mater.* **27**, 1090–1096 (2015).

10. Lin, H. H. Mock, J. Smith, D. Gao, T. & Sailor, M. J. Surface-enhanced Raman scattering from silver-plated porous silicon. *J. Phys. Chem. B*, **108***,* 11654–11659 (2004).

11. Liang, H. Y. et al. Highly surface-roughened "flower-like" silver nanoparticles for extremely sensitive substrates of surface-enhanced Raman scattering. *Adv. Mater.* **21**, 4614–4618 (2009).

12. Das, G. Chakraborty, R. Gopalakrishnan, A. Baranov, D. Fabrizio, E. D. & Krahne, R. A new route to produce efficient surface-enhanced Raman spectroscopy substrates: gold-decorated CdSe nanowires. *J Nanopart Res*. **15**, 1596 (2013).

13. Wang, Y. F. et al. Mercaptopyridine Surface-Functionalized CdTe Quantum Dots with Enhanced Raman Scattering Properties. *J. Phys. Chem. C*. **112**, 996–1000 (2008).

14. Wang, Y. F. Sun, Z. H. Wang, Y. X. Hu, H. L. Zhao, B. Xu, W. Q. Lombardi, J. R. Surface-enhanced Raman scattering on mercaptopyridine-capped CdS microclusters *Spectrochimica*. *Acta*. *Part*. *A*. **66**, 1199–1203 (2007).

15. Wen, H. HE, T.J. XU, C.Y. ZUO, J. LIU, F. C. Surface enhancement of Raman and absorption spectra from cyanine dye D266 adsorbed on ZnO colloids. *Molecular Physics*. **88**, 281–290 (1996).

16. Qi, D. Lu, L. Wang, L. Zhang, J. Improved SERS sensitivity on plasmon-free

TiO_2 photonic microarray by enhancing light-matter coupling. *J. Am. Chem. Soc.***136**, 9886-9889 (2014).

17. Zou, X. X. Silva, R. Huang, X. X. Al-Sharabc, J. F. Asefa, T. A self-cleaning porous TiO_2–Ag core–shell nanocomposite material for surface-enhanced Raman scattering. *Chem. Commun.* **49**, 382 (2013).

18. Fu, X. Q. Bei, F. L. Wang, X. Yang, X. J. Lu, L. D. Surface-enhanced Raman scattering of 4-mercaptopyridine on sub-monolayers of α-Fe_2O_3 nanocrystals (sphere, spindle, cube). *J. Raman Spectrosc.* **40**, 1290–1295 (2009).

19. Jiang, L. You, T. Yin, P. Shang, Y. Zhang, D. Guo, L. Yang, S. Surface-enhanced Raman scattering spectra of adsorbates on Cu_2O nanospheres: charge-transfer and electromagnetic enhancement. *Nanoscale* **5**, 2784–2789 (2013).

20. Wang, Y. Hu, H. Jing. S. Wang, Y. Sun, Z. Zhao, B. Zhao, C. Lombardi, J. R. Enhanced Raman scattering as a probe for 4-mercaptopyridinesurface-modified copper oxide nanocrystals. *Anal. Sci.* **23**, 787–791 (2007).

21. Cong, S. et al. Noble metal-comparable SERS enhancement from semiconducting metal oxides by making oxygen vacancies. *Nat. Commun*. **6**, 7800 (2015).
